# Lack of Serologic Evidence to Link IgA Nephropathy with Celiac Disease or Immune Reactivity to Gluten

**DOI:** 10.1371/journal.pone.0094677

**Published:** 2014-04-14

**Authors:** Sina Moeller, Pietro A. Canetta, Annette K. Taylor, Carolina Arguelles-Grande, Holly Snyder, Peter H. Green, Krzysztof Kiryluk, Armin Alaedini

**Affiliations:** 1 Division of Digestive and Liver Diseases, Department of Medicine, Columbia University Medical Center, New York, New York, United States of America; 2 Celiac Disease Center, Columbia University Medical Center, New York, New York, United States of America; 3 Division of Nephrology, Department of Medicine, Columbia University Medical Center, New York, New York, United States of America; 4 Esoterix, Inc., Laboratory Corporation of America Holdings, Englewood, Colorado, United States of America; 5 Institute of Human Nutrition, Columbia University Medical Center, New York, New York, United States of America; Wadsworth Center, New York State Dept. Health, United States of America

## Abstract

IgA nephropathy is the most common form of primary glomerulonephritis worldwide. Mucosal infections and food antigens, including wheat gluten, have been proposed as potential contributing environmental factors. Increased immune reactivity to gluten and/or association with celiac disease, an autoimmune disorder triggered by ingestion of gluten, have been reported in IgA nephropathy. However, studies are inconsistent about this association. We aimed to evaluate the proposed link between IgA nephropathy and celiac disease or immune reactivity to gluten by conducting a comprehensive analysis of associated serologic markers in cohorts of well-characterized patients and controls. Study participants included patients with biopsy-proven IgA nephropathy (n = 99), unaffected controls of similar age, gender, and race (n = 96), and patients with biopsy-proven celiac disease (n = 30). All serum specimens were tested for IgG and IgA antibodies to native gliadin and deamidated gliadin, as well as IgA antibody to transglutaminase 2 (TG2). Anti-TG2 antibody-positive nephropathy patients and unaffected controls were subsequently tested for IgA anti-endomysial antibody and genotyped for celiac disease-associated HLA-DQ2 and -DQ8 alleles. In comparison to unaffected controls, there was not a statistically significant increase in IgA or IgG antibody reactivity to gliadin in individuals with IgA nephropathy. In addition, the levels of celiac disease-specific serologic markers, i.e., antibodies to deamidated gliadin and TG2, did not differ between IgA nephropathy patients and unaffected controls. Results of the additional anti-endomysial antibody testing and HLA genotyping were corroborative. The data from this case-control study do not reveal any evidence to suggest a significant role for celiac disease or immune reactivity to gluten in IgA nephropathy.

## Introduction

Immunologic sensitivity to dietary gluten (comprised of gliadin and glutenin proteins) from wheat and related cereals is most extensively studied and best understood in the context of celiac disease and wheat allergy [1]. In celiac disease, the ensuing innate and adaptive immune responses to ingested gluten lead to inflammation and villous atrophy in the small intestine, although the condition is also known to have a number of extra-intestinal manifestations [2]. Genetic susceptibility in celiac disease is closely linked to genes for class II human leukocyte antigens (HLA) DQ2 and DQ8 [3]. The associated humoral immune response includes antibodies to native and deamidated sequences of gliadin, as well as autoantibodies against endomysial tissue, the primary target of which is the transglutaminase 2 (TG2) enzyme [4]. IgA anti-TG2 (or anti-endomysial) autoantibody is considered to be the most specific and sensitive serologic marker of the condition, being used widely to aid diagnosis [5]. Some individuals, despite lacking the required serologic, histologic, or genetic markers of celiac disease and wheat allergy, experience intestinal or extra-intestinal symptoms in response to ingestion of wheat, sometimes in conjunction with elevated antibody reactivity to native gliadin [6]. The term “non-celiac gluten sensitivity” has been proposed to refer to the spectrum of symptoms reported by these patients [1], although a role for gluten as the specific culprit has not been well established [6].

Primary IgA nephropathy (IgAN) is the most common form of primary glomerulonephritis. IgAN patients present with microscopic or intermittent macroscopic hematuria, varying degrees of proteinuria, and often develop renal insufficiency [7]. There is no disease-targeted treatment for IgAN and the factors that lead to disease development are poorly understood. A hallmark of IgAN pathogenesis is a dysregulation in the synthesis and metabolism of IgA that favors formation of IgA-containing immune complexes and their subsequent mesangial deposition [7], [8]. Although several recent studies highlight the contribution of genetic factors in the pathogenesis of IgAN, environmental exposures, including immunogenic molecules of microbial or food origin have also been suggested to play a role [9]. Ingestion of wheat gluten has been reported to induce IgA mesangial deposits in BALB/c mice in one study [10]. There are also case reports indicating positive outcome or alteration of certain immune abnormalities in response to gluten restriction in IgAN [11]–[13]. Of particular note, elevated immune reactivity to wheat gluten has been reported to be associated with IgAN in several studies, with rates of 30–50% or greater positivity for IgA antibodies to gliadin proteins in affected patients[14]–[16]. Some studies have also described increased frequency of markers that have greater specificity for celiac disease, including anti-endomysial antibodies, or of biopsy-proven celiac disease, in the context of IgAN [17], [18]. Reports from other investigators have challenged these findings [19]–[21]. Overall, these studies have been limited by small sample sizes, lack of suitable control groups, and/or technical issues regarding assay methodology, rendering them inconclusive. In the current study, we evaluated the proposed connection between IgAN and celiac disease or immune reactivity to gluten through comprehensive analysis of associated serologic markers in a comparatively large and well-characterized case-control population.

## Methods

### Patients and Controls

Study participants included 99 patients with IgAN and 96 unaffected controls of similar age, gender, and race, all of them residing in New York or New Jersey. All cases carried a biopsy diagnosis of IgAN, defined by typical light microscopy features and predominant IgA staining on kidney tissue immunofluorescence in the absence of liver disease or other autoimmune conditions. Disease duration, for all patients and regardless of whether they were eventually transplanted, was calculated as the time from initial diagnosis made on native kidney biopsy. Screening questionnaires were used to evaluate the general health of unaffected controls. Individuals who reported having a history of renal disease, proteinuria, or hematuria were excluded. Fifty-four (56%) of the unaffected controls were also screened by urinalysis and were negative for hematuria or proteinuria. Additional controls included 30 patients with biopsy-proven celiac disease, diagnosed according to previously described criteria [2]. Written informed consent was obtained for all study participants. This study was approved by the Institutional Review Board of Columbia University Medical Center. Serum and DNA samples were kept at −80°C to maintain stability.

### Gliadin

The antigen used for the anti-gliadin antibody assays was the Prolamine Working Group (PWG) reference gliadin, which was extracted from a combination of 28 different wheat varieties, as previously described [22]. The protein profile of the PWG gliadin extract was assessed by SDS-polyacrylamide gel electrophoresis, using 10% NuPAGE Bis-Tris precast gels and 3-(N-morpholino)propanesulfonic acid (MOPS) buffer (Life Technologies, Carlsbad, Calif.).

### Anti-gliadin Antibodies

Serum IgA and IgG antibodies to gliadin were measured by enzyme-linked immunosorbent assay (ELISA) as previously described [23], [24], with minor modifications. A 2 mg/mL stock solution of the PWG gliadin was prepared in 60% ethanol. 96-well Maxisorp round-bottom polystyrene plates (Nunc, Roskilde, Denmark) were coated with 50 µL/well of a 0.01 mg/mL solution of gliadin in 0.1 M carbonate buffer (pH 9.6) or were left uncoated to serve as control wells. After incubation at 37°C for 1 h, all wells were washed and blocked by incubation with 1% bovine serum albumin (BSA) in phosphate buffered saline containing 0.05% Tween-20 (PBST) for 1.5 h at room temperature. Serum samples were diluted at 1∶800 for IgG measurement and at 1∶200 for IgA measurement, added at 50 µL/well in duplicates, and incubated for 1 h. Each plate contained a positive control sample from a patient with biopsy-proven celiac disease and elevated IgG and IgA antibodies to gliadin. After washing the wells, they were incubated with HRP-conjugated anti-human IgG (GE Healthcare, Piscataway, N.J.) or IgA (MP Biomedicals, Santa Ana, Calif.) secondary antibodies for 50 min. The plates were washed and 50 µL of developing solution, comprising of 27 mM citric acid, 50 mM Na_2_HPO_4_, 5.5 mM *o*-phenylenediamine, and 0.01% H_2_O_2_ (pH 5), was added to each well. After incubating the plates at room temperature for 20 min, absorbance was measured at 450 nm. Absorbance values were corrected for non-specific binding by subtraction of the mean absorbance of the associated BSA-coated control wells. The corrected values were first normalized according to the mean value of the positive control duplicate on each plate. The mean antibody level for the healthy control cohort was then set as 1.0 AU and all other results were normalized to this value.

### Anti-deamidated Gliadin Antibodies

IgA and IgG antibody reactivities to a previously described glutamine-glutamate substituted trimer of a fusion peptide, containing the sequences PLQPEQPFP and PEQLPQFEE [25], were measured by separate ELISAs, according to the manufacturer’s protocols (Euroimmun AG, Lubeck, Germany).

### Anti-transglutaminase 2 (TG2) Antibody

IgA antibody to recombinant human TG2 was measured by ELISA, according to the manufacturer’s protocol (Euroimmun AG).

### Anti-endomysial Antibody

Sera from anti-TG2 antibody-positive subjects were tested in duplicate for anti-endomysial antibody reactivity by an indirect immunofluorescence assay, using the NOVA Lite Monkey Esophagus IFA Kit/Slides (Inova, San Diego, Calif.), according to the manufacturer’s instructions.

### HLA Genotyping

High resolution HLA-DQA1 and -DQB1 genotyping of anti-TG2 antibody-positive subjects was performed by DNA amplification through multiplex polymerase chain reaction (PCR), followed by detection with sequence-specific oligonucleotide (SSO) probes [26]. Extracted DNA was amplified and hybridized to a suspension of fluorescent beads containing immobilized DNA probes specific to DQA1 and DQB1 polymorphic sequences, according to the kit manufacturer’s instructions (One Lambda, Canoga Park, Calif.). The hybridized beads were analyzed on a LABScan 100 platform, utilizing Luminex technology, and interpreted using software provided by the manufacturer (One Lambda). Presence or absence of celiac disease-associated DQA1*0501/0505-DQB1*0201/0202 (DQ2) and DQA1*03-DQB1*0302 (DQ8) genes was determined.

### Data Analysis

Differences between groups were analyzed by the one-way analysis of variance (ANOVA) with post-hoc testing for multiple comparisons (continuous data), and by the Fisher’s exact test (nominal data). Adjustment for covariate effect (age, gender, and race) was carried out by analysis of covariance (ANCOVA), using the general linear model. Elevated levels of anti-gliadin antibody were defined as values at the 95th percentile or higher in the healthy control group. For IgA anti-TG2 antibody and IgA/IgG anti-deamidated gliadin antibodies, cutoffs for positivity were assigned by the manufacturer (1.0 AU). Differences with *p* values of *<*0.05 were considered to be statistically significant. Statistical analyses were performed with Prism 6 (GraphPad, San Diego, Calif.) and Minitab 16 (Minitab, State College, Pa.).

## Results

### Patients and Controls

The demographic and clinical characteristics of the patients and controls are shown in Table 1. There was no significant difference between the two groups with regard to age, gender, or race. Among the IgAN patients, 49% were receiving immunosuppressants at the time of sampling, including 30 on mycophenolate (alone or in combination with other agents), 14 on glucocorticoid monotherapy, and 2 on combination cyclophosphamide/glucocorticoid. In addition, 79 (80%) were being treated with angiotensin-converting enzyme inhibitor (ACEI), angiotensin receptor blocker (ARB), or both.

**Table 1:** Demographic and clinical characteristics of study cohorts.

Study group	Mean age–years±SD	Male sex–no.(%)	White race–no.(%)	Disease duration–years±SD	Post-kidneytransplant–no.(%)	Immunosuppressivetherapy–no. (%)	ACEI or ARBtherapy–no. (%)^1^
**IgAN (n = 99)**	42.3**±**13.4	63 (64)	98 (99)	7.3**±**7.3	28 (28)	49 (50)	79 (80)
**Unaffected** **control (n = 96)**	43.8**±**12.7	55 (57)	94 (98)	**–**	**–**	**–**	**–**
**Celiac disease (n = 30)**	46.9**±**16.2	11 (37)	30 (100)	**–**	**–**	**–**	**–**

1 ACEI: Angiotensin-converting enzyme inhibitor; ARB: angiotensin receptor blocker.

### Gliadin

**Figure 1:**
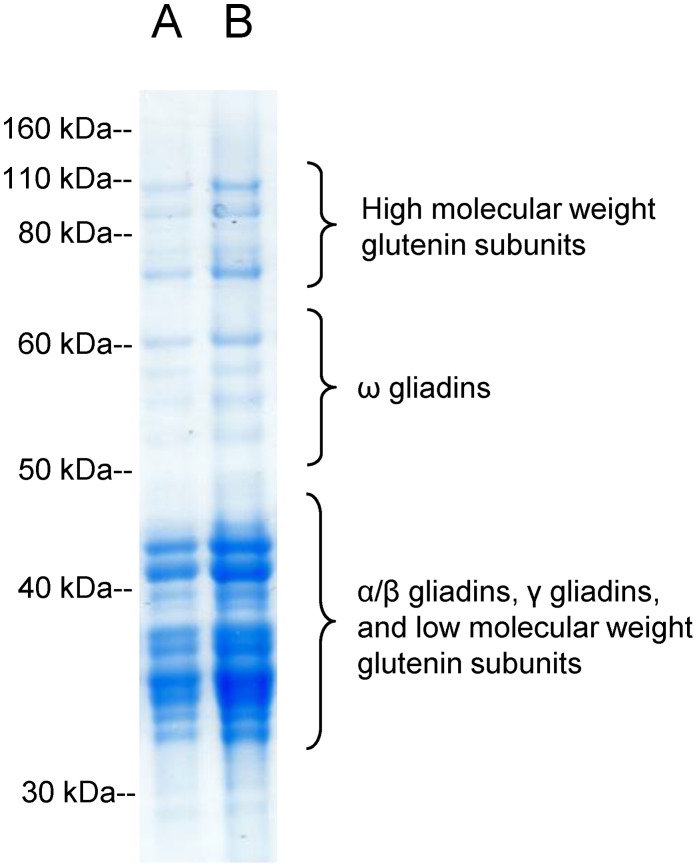
Gel electrophoresis profile of the gliadin preparation used for the anti-gliadin antibody assays. A) 5 µg of protein loaded; B) 10 µg of protein loaded.

The gel electrophoresis profile of the PWG gliadin used for the anti-gliadin antibody assays indicated the presence of all main types of gliadin proteins, α/β, γ, and ω. The mixture also contained high and low molecular weight glutenin subunits (Fig. 1).

### Antibody Measurements and HLA Genotyping

**Figure 2:**
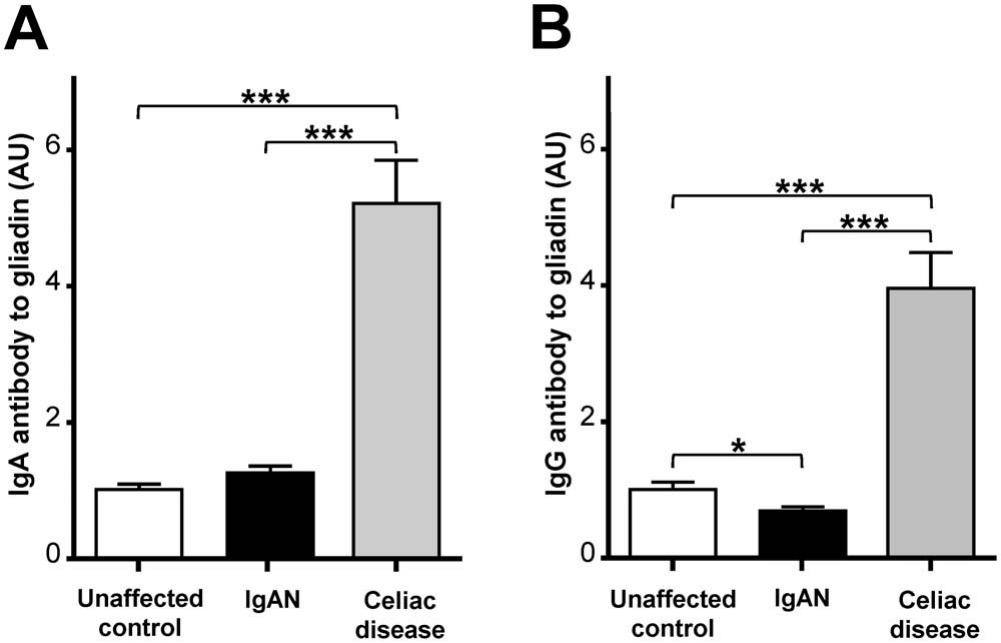
Mean levels of A) IgA and B) IgG antibody to gliadin in IgAN patients and unaffected controls, as well as individuals with celiac disease. Error bars represent the standard error of the mean. * = *p*<0.05, *** = *p*<0.001.

**Figure 3:**
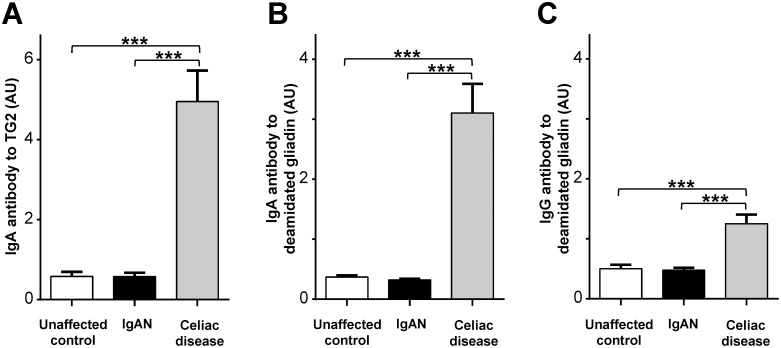
Mean levels of A) IgA anti-human TG2, B) IgA anti-deamidated gliadin, and C) IgG anti-deamidated gliadin in IgAN patients and unaffected controls, as well as individuals with celiac disease. Error bars represent the standard error of the mean. *** = *p*<0.001.

Mean levels of IgA and IgG class antibodies to gliadin and deamidated gliadin, as well as IgA antibody to TG2, for patient and control groups are presented in Fig. 2 and Fig. 3. The IgAN patient cohort exhibited slightly higher mean IgA antibody reactivity to gliadin when compared with the unaffected control group, but the difference was not statistically significant (*p* = 0.11) (Fig. 2). IgAN patients had lower levels of IgG antibody to gliadin in comparison to unaffected controls and the difference remained significant after adjusting for the covariates of age, gender, and race (*p*<0.05) (Fig. 2). There were no differences in levels of IgA antibody to TG2 (*p* = 0.60), IgA antibody to deamidated gliadin (*p* = 0.45), or IgG antibody to deamidated gliadin (*p* = 0.99) between IgAN patients and unaffected controls. Patients with celiac disease exhibited significantly greater levels of IgA and IgG antibodies to gliadin and deamidated gliadin, as well as IgA antibody to TG2, when compared to IgAN patients or unaffected controls (*p*<0.001 for all comparisons).

There were no significant differences in the frequencies of any of the examined serologic markers between the IgAN cohort and the unaffected control group. Specifically, 8/99 (8%) of IgAN patients and 4/96 (4%) of unaffected controls were positive for IgA anti-gliadin antibody (*p* = 0.4), whereas 1/99 (1%) of IgAN patients and 4/96 (4%) of unaffected controls were positive for IgG anti-gliadin antibody (*p* = 0.2). Three (3%) IgAN patients and 3 (3%) unaffected controls were positive for IgA anti-TG2 antibody (*p* = 0.9). Of these, 1 IgAN patient and 1 control had values that were near the cutoff and therefore equivocal. Eight (8%) IgAN patients and 4 (4%) unaffected controls were positive for IgA anti-deamidated gliadin antibody (*p* = 0.4), while 1 (1%) IgAN patients and 4 (4%) unaffected controls were positive for IgG anti-deamidated gliadin antibody (*p* = 0.2).

In order to further assess the likelihood of celiac disease in the 3 IgAN patients and 3 unaffected controls who were anti-TG2 antibody-positive, genotyping for celiac disease-associated HLA-DQ2/DQ8 alleles and testing for IgA anti-endomysial antibody reactivity were performed. Three of 3 IgAN patients and 2 of 3 unaffected controls were HLA DQ2- or DQ8-positive, while 1 unaffected control was positive for one half of the DQ2 heterodimer (Table 2). These genotypes are all consistent with celiac disease. Two of 3 IgAN patients and 2 of 3 controls were positive for anti-endomysial antibody, corresponding to the same individuals that were unequivocally positive for anti-TG2 antibody (Table 2). These 2 patients and 2 unaffected controls are likely to have celiac disease.

**Table 2:** Antibody and HLA data for the anti-TG2 antibody-positive patients and controls.

Subjects	Anti-TG2 antibody level (AU)^1^	Anti-endomysial antibody reactivity^2^	HLA^3^
**IgAN patient 1**	1.25	Negative	DQ2
**IgAN patient 2**	6.17	Positive	DQ8
**IgAN patient 3**	10.0	Positive	DQ2
**Unaffected control 1**	1.06	Negative	DQ2
**Unaffected control 2**	7.72	Positive	½ DQ2 (DQA1*0501/0505)
**Unaffected control 3**	9.90	Positive	DQ8

1 Measured by ELISA (cutoff = 1.0).

2 Detected by indirect immunofluorescence assay.

3 DQ2 = DQA1*0501/0505-DQB1*0201/0202; DQ8 = DQA1*03-DQB1*0302.

A significant correlation between antibody levels and duration of disease was not found in the IgAN patient cohort. There was no significant difference for any of the antibody markers based on treatment status.

## Discussion

The data from this study indicate that, compared with unaffected controls, neither antibodies to native gliadin, nor the more specific and sensitive markers of celiac disease, i.e., antibodies to TG2 and deamidated gliadin, are significantly elevated in patients with IgAN. Furthermore, the observed incidence of unequivocal positivity for anti-TG2 and anti-endomysial antibodies points to a frequency of celiac disease in the IgAN and unaffected control groups that is similar to that reported for the general population [27]–[29]. The concomitant existence of slightly increased IgA and decreased IgG reactivity to gluten in the IgAN cohort compared with unaffected controls is likely to be related to the systemic IgA-restricted B cell hyperactivity. Earlier studies on B cells from IgAN patients have shown that the antibody response to antigenic stimulation is primarily geared towards IgA synthesis, with an apparent decline in IgG release [30], [31].

A recently published large epidemiological study from Sweden investigated whether IgAN occurs more commonly in patients with celiac disease than in reference individuals [32]. Despite the remarkably large sample size for each group, the study found an increase in the rate of IgAN among patients with celiac disease compared with reference individuals that was marginally significant, possibly attributable to the inherent bias towards further evaluation and follow-up of individuals already diagnosed with a chronic disease. The Swedish study also demonstrated a low absolute risk of developing IgAN, with only 0.026% of celiac disease patients being diagnosed later with the condition. While the two studies are different in objective and methodology, the Swedish epidemiologic data and the serologic analysis by our group are confirmatory in that they show the co-occurrence of celiac disease and IgAN to be a rare event.

Although the current study is the largest and most comprehensive analysis of serologic markers of celiac disease and immune reactivity to gluten in IgAN to date, it does have some limitations. First, it is a single-center study, based primarily on individuals identified in their records as being white and residing in the northeastern United States. Thus our findings may not be generalizable to other races or geographic locations. In addition, we could not control for the diet of the research participants, which may contribute to levels of antibodies against gliadin and other antigens in patients and controls. Finally, duodenal biopsy, which is regarded as the gold standard for diagnosis of celiac disease, was not part of this study. However, considering the excellent sensitivity and specificity of anti-TG2 (and to a somewhat lesser extent anti-deamidated gliadin) antibodies [5], it can be concluded with high certainty that the patients with normal serology do not have celiac disease.

In conclusion, the data from this study do not provide any evidence to suggest frequent co-occurance of IgAN with celiac disease or with increased immune reactivity to gluten. It should be noted that the results do not address whether immune reactivity to gluten can ever be a contributing factor for IgAN. However, the study’s sample size is large enough to effectively rule out the suggested high rates of elevated antibody to gluten among IgAN patients. Celiac disease and immune reactivity to gluten are not likely to be significant players in the pathophysiology of IgAN.
